# The clinical and radiological outcomes of hip resurfacing versus total hip arthroplasty: a meta-analysis and systematic review

**DOI:** 10.3109/17453674.2010.533933

**Published:** 2010-11-26

**Authors:** Toby O Smith, Rachel Nichols, Simon T Donell, Caroline B Hing

**Affiliations:** ^1^Faculty of Health, University of East Anglia, Norwich; ^2^Physiotherapy Department, Dereham Hospital, Norfolk PCT, Norwich; ^3^Institute of Orthopaedics, Norfolk and Norwich University Hospital, Norwich; ^4^Department of Trauma and Orthopaedics, St. George's Hospital, London, UK

## Abstract

**Background and purpose:**

Hip resurfacing (HRS) procedures have gained increasing popularity for younger, higher-demand patients with degenerative hip pathologies. However, with concerns regarding revision rates and possible adverse metal hypersensitivity reactions with metal-on-metal articulations, some authors have questioned the hypothesized superiority of hip resurfacing over total hip arthroplasty (THA). In this meta-analysis, we compared the clinical and radiological outcomes and complication rates of these 2 procedures.

**Methods:**

A systematic review was undertaken of all published (Medline, CINAHL, AMED, EMBASE) and unpublished or gray literature research databases up to January 2010. Clinical and radiological outcomes as well as complications of HRS were compared to those of THA using risk ratio, mean difference, and standardized mean difference statistics. Studies were critically appraised using the CASP appraisal tool.

**Results:**

46 studies were identified from 1,124 citations. These included 3,799 HRSs and 3,282 THAs. On meta-analysis, functional outcomes for subjects following HRS were better than or the same as for subjects with a THA, but there were statistically significantly greater incidences of heterotopic ossification, aseptic loosening, and revision surgery with HRS compared to THA. The evidence base showed a number of methodological inadequacies such as the limited use of power calculations and poor or absent blinding of both patients and assessors, possibly giving rise to assessor bias.

**Interpretation:**

On the basis of the current evidence base, HRS may have better functional outcomes than THA, but the increased risks of heterotopic ossification, aseptic loosening, and revision surgery following HRS indicate that THA is superior in terms of implant survival.

Over the last 3 decades, the threshold for total hip arthroplasty (THA) has been lowered to include younger and more physically demanding patients (Abraham et al. 2006, Treacy 2006). However, hip arthroplasty surgery in younger patients with more demanding lifestyles has been reported to fail earlier (Tennent and Goddard 2000, Lewthwaite et al. 2008, Howcroft et al. 2008). In order to address this issue, some surgeons have advocated the use of metal-on-metal or ceramic bearing surfaces with cementless porous coated prostheses to reduce implant surface wear rates and bone loss in younger osteoarthritics (Shetty and Villar 2006). Other surgeons have advocated hip resurfacing (HRS) for young patients (Harty et al. 2005, Abraham et al. 2006, Bengs et al. 2008, Greene et al. 2009).

Hip resurfacing has gained wider support over the past 10 years with the development of more successful implants and improvements in manufacturing techniques and materials compared to previous generations of failed HRS designs (Treacy 2006, Deuel et al. 2009). Hip resurfacing theoretically allows for greater bone stock preservation, lower wear rates, retention of the femoral neck, and the use of a larger bearing surface (McMinn and Daniel 2006, Hing et al. 2007, Heilpern et al. 2008, Steffen et al. 2008, Daniel et al. 2010). Some authors have suggested that approximation to the native femoral head-to-neck ratio therefore allows greater range of motion before component impingement, and improved function over conventional THA (Chandler et al. 1982, Alberton et al. 2002, Barrack 2003). Proponents of HRS have also suggested that if revision surgery is required, converting to a THA is easier than revising a THA and is theoretically similar to performing a primary THA, due to the greater bone preservation initially (Ball et al. 2007, Bengs et al. 2008). However, concerns have been raised recently regarding systemic exposure to cobalt and chromium ions, with the larger bearings used in HRS resulting in aseptic lymphocytic vasculitis-associated lesions (ALVAL); the long-term consequences of this are yet to be defined (Davies et al. 2005, Pandit et al. 2008, Glyn-Jones et al. 2009, Ollivere et al. 2009). Furthermore, some authors have also suggested that the proposed advantages of HRS with respect to range of motion and functional outcomes may not be true compared to contemporary THA surgery (Bengs et al. 2008, Le Duff et al. 2009, Malviya et al. 2010). In addition, revision of an HRS may in fact be more technically demanding than that of a primary THA (Günther et al. 2008, Taylor et al. 2009).

Given the debate about the efficacy of these 2 implant designs, we wanted to determine whether there is a difference in clinical and radiological outcomes between conventional THA and HRS. While previous studies have narratively reviewed the evidence base on this topic or compared the clinical outcomes of THA and HRS cohorts from separate studies (Wyness et al. 2004, Marker et al. 2009, Springer et al. 2009), there has been no formal meta-analysis comparing THA and HRS cohorts after a systematic review.

## Materials and methods

### Search strategy

All searches were conducted on January 10, 2010. The primary search was of the databases Medline (1950 to January 2010), CINAHL (1982 to 1950 to January 2010), AMED (1985 to 1950 to January 2010) and EMBASE (1974 to 1950 to January 2010). These were searched via Ovid using MeSH terms and the Boolean operators “hip AND (replacement OR arthroplasty) AND resurfacing”. A secondary search of unpublished literature was conducted using the databases SIGLE (System for Information on Grey Literature in Europe), the National Technical Information Service, the National Research Register (UK), the British Library's Integrated Catalogue, and Current Controlled Trials using the same search terms as used in the primary search. Broad search terms were used to minimize the possibility of omitting important citations from the review. Conference proceedings of the British Orthopaedic Association (BOA) Annual Congress, the European Federation of National Associations of Orthopaedics and Traumatology (EFORT), and the British Hip Society were searched from their inception to January 2010. The reference lists of each potentially relevant paper and review papers were appraised for relevant papers not identified by the initial search. Finally, the corresponding authors of each paper included were contacted for citations not identified from the original searches.

### Eligibility criteria

Using the results from the search strategy, all randomized controlled trials (RCTs) and non-randomized controlled trials (nRCTs) comparing HRS and THA implants for patients with hip pathology were identified and included. The search strategy was unspecific regarding the joint prostheses used for each cohort, subject age, sex, and the rationale for surgery. There were no language restrictions in the searches. Animal studies, cadaver studies, single case reports, comments, letters, editorials, protocols, guidelines, publications based on surgical registries, and review papers were excluded due to their methodological quality. 2 reviewers (TS and RN) independently reviewed the eligibility of each citation identified, using the titles and abstracts based on these criteria. For each eligible or potentially eligible article, full text versions were ordered when available.

### Data extraction

Data were extracted from the included papers by 1 reviewer and verified by a second review using a predefined data extraction spreadsheet. Data fields extracted included: operative techniques, study sample size, cohort age at surgery, sex, indications for surgery, implants used, assessment procedures and outcome measures, results, and follow-up period.

### Critical appraisal

All the papers included were independently assessed by 2 reviewers using a modified CASP assessment tool (CASP 2010). This is a 17-item appraisal tool consisting of 4 sections: an assessment of study validity; an evaluation of methodological quality such as subject identification, randomization, blinding, and subject drop-out rates; an assessment of the presentation of results using descriptive and inferential statistics with confidence intervals; and an assessment of external validity and generalizability to clinical practice.

Any disagreements about paper eligibility, data extraction results, or critical appraisal score were resolved through discussion between the independent reviewers.

### Primary outcome measure

The primary outcome measure was frequency of revision surgery.

### Secondary outcome measures

Secondary clinical outcome measures included: incision length, last acetabular reamer size, duration of operation, blood loss and frequency of blood transfusion requirement, length of hospital stay, pain, functional outcome and quality of life outcome, and hip range of motion. Radiological outcomes included: femoral and acetabular offset, the frequency of femoral or acetabular radiolucency, leg length, cup height (measured as the distance in the vertical plane from the center of rotation of the acetabulum to the line drawn between the base of the teardrops, parallel to Hilgenreiner's line (Loughead et al. 2005), and the incidence of heterotopic ossification. Complications assessed included: venous thromboembolic events (VTEs), acetabular component malposition, trochanteric malunion or nonunion, nerve palsy, presence of a Trendelenburg sign, fracture incidence and femoral neck notch incidence, dislocation rate, incidence of aseptic loosening or avascular necrosis, infection, and mortality.

### Review protocol

A review protocol was not published before commencing the study.

### Statistics

A meta-analysis was undertaken using the results from the agreed extraction table. Meta-analysis was only conducted where there was no observed evidence of a substantial difference in study populations, interventions, or outcome measures on review of the extraction table. We assessed statistical heterogeneity with Chi^2^ and I^2^ statistical tests. Where statistical heterogeneity (measured using I^2^) was less than 20%, a fixed effects model was used. For outcomes above 20%, a random effects model was used (Higgins et al. 2003). The Mantel-Haenszel method was used to calculate mean pooled difference (MD) for continuous data, and pooled risk ratios (RR) for dichotomous data (Mantel and Haenszel 1959). A probability of p < 0.05 was regarded as statistically significant, and 95% confidence intervals (CIs) were calculated. When not enough data were available in the original report or publication, attempts were made to contact the corresponding authors. Finally, a funnel plot was generated to assess publication bias for the outcome measure most frequently reported.

The meta-analysis was conducted by one investigator using REVMAN software (version 5.0 for Windows; the Nordic Cochrane Center, Copenhagen, Denmark) (The Cochrane Collaboration, 2008).

## Results

### Search strategy

**Figure 1.**
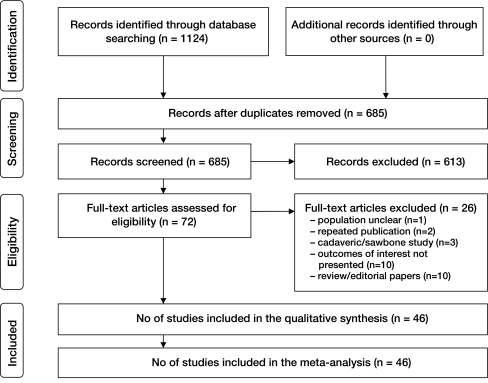
PRISMA flow chart.

**Figure 2.**
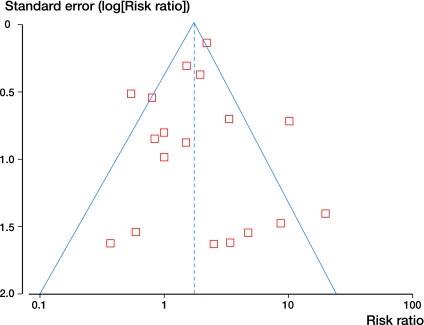
Funnel plot showing limited evidence of publication bias for the frequency of revision surgery.

1,124 citations were identified from the search strategy. 46 studies were deemed appropriate (Figure 1). The findings of one study appeared to be reported in 2 papers (Vendittoli et al. 2006a, b). We therefore included all data from the publication which presented the largest dataset (Vendittoli et al. 2006a) and excluded the other publication. As Figure 2 shows, there was minimal publication bias evident for the primary outcome frequency of revision surgery.

### Cohort characteristics

From the 46 citations, 28 prospective observational studies, 8 retrospective studies, and 10 RCTs were identified (Tables 1–3). 3,799 HRSs in 3,279 patients were compared to 3,282 THAs in 2,910 patients. Mean age in the HRS group was 51 (SD 7) years, while mean age in the THA group was 54 (SD 8) years. There was a trend of an older average age of subjects in the THA groups compared to HRS groups (see Tables 1–3). In the HRS cohorts, 1,578 males were compared to 806 females; 7 papers did not state the sex of the patients. In the THA cohorts, 1,176 males were compared to 959 females, and 8 papers did not state the sex of the patients. Mean follow-up period was 25 (SD 27) months, as stated in 23 studies. This ranged from immediately postoperatively (Vendittoli et al. 2006a, Brennan et al. 2009) to 82 months (Gustilo et al. 1983).

**Table 1.** Demographic characteristics of randomized controlled trials

Paper	Hips		Patients		Mean age		Gender (M/F)		Follow-up period
	HRS	THA	HRS	THA	HRS	THA	HRS	THA	(months)
Garbuz et al. (2010)	48	56	48	56	52	52	43/5	50/6	Min. 12
Girard et al. (2008)	69	76	69	76	50	50	38/31	48/28	21
Girard et al.(2006)	49	55	49	55	47	48	31/18	34/21	N/S
Howie et al. (2005)	11	13	11	13	46	50	6/5	9/4	24
Lavigne et al. (2009)	24	24	24	24	50	50	14/10	5/9	14
Lavigne et al. (2008)	81	71	81	71	48	50	53/28	51/20	27
Rama et al. (2009)	103	97	103	97	50	50	65/38	66/31	12
Vendittoli et al. (2006)	107	103	107	103	49	51	67/40	70/33	Intra-op.
Vendittoli et al. (2010a)	64	53	64	53	49	51	42/22	33/20	24
Vendittoli et al. (2010b)	109	100	109	100	49	51	69/40	68/32	24

Intra-op.: intra-operatively; N/S: not stated; THA: total hip replacement.

**Table 2.** Demographic characteristics of retrospective studies

Paper	Hips		Patients		Mean age		Gender (M/F)		Follow-up period
	HRS	THA	HRS	THA	HRS	THA	HRS	THA	(months)
Brennan et al. (2009)	31	31	31	31	59	61	24/7	13/18	Intra-op.
Kim et al. (1987)	106	98	N/S	N/S	27	28	65/41	56/42	60
Meldrum et al. (2008)	141	125	118	103	47	47	80/32	58/45	Min. 14
Mont et al. (2009)	54	54	54	54	55	55	36/18	36/18	39
Naal et al. (2009)	362	181	N/S	N/S	N/S	N/S	250/112	125/56	Intra-op.
Pollard et al. (2006)	54	51	54	53	50	50	40/11	40/13	Min. 71
Swank and Alkire (2009)	128	105	128	105	51	N/S	100/28	N/S	24
Vail et al. (2006)	57	93	52	84	47	57	41/11	23/61	35

Intra-op.: intra-operatively; N/S: not stated; THA: total hip replacement.

**Table 3.** Demographic characteristics of observational studies

Paper	Hips		Patients		Mean age		Gender (M/F)		Follow-up period
	HRS	THA	HRS	THA	HRS	THA	HRS	THA	(months)
Ahmad et al. (2009)	28	28	28	28	54	61	14/14	10/18	N/S
Amstitz et al. (1984)	100	100	91	86	58	66	60/31	35/51	Min. 22
Chirodian et al. (2004)	44	50	44	50	48	52	28/16	22/28	12
Fowble et al. (2009)	50	44	50	35	46	55	31/19	18/26	Min. 24
Gore et al. (1985)	27	29	27	29	55	62	27	29	N/S
Gustilo et al. (1983)	75	91	67	75	75	49	N/S	N/S	82
Hall et al. (2009)	33	99	33	99	54	55	27/6	81/18	6
Hayaishi et al. (2007)	10	16	10	16	N/S	N/S	N/S	N/S	12
Kishida et al.(2004)	13	13	13	12	58	58	7/6	3/9	24
Langton et al. (2010) BHR	155	87	155	87	51	67	88/67	34/53	Min. 8
Langton et al. (2010) ASR	418	87	418	87	56	67	234/184	34/53	Min. 8
Le Duff et al. (2009)	35	35	35	35	53	53	24/11	24/11	88
Leonard et al. (2007)	40	85	40	85	N/S	N/S	N/S	N/S	N/S
Lilikakis et al. (2005)	35	41	33	40	53	65	20/13	14/26	0.3
Lingard et al. (2009)	132	214	132	214	49	67	90/42	90/146	12
Loughead et al. (2005)	28	26	28	26	50	62	N/S	N/S	N/S
Mont et al. (2007)	15	15	15	15	51	58	10/5	9/6	12
Mont et al. (2001)	30	30	30	30	34	35	18/12	18/12	84
Moroni et al. (2008)	25	27	20	26	49	48	10/10	12/14	Min. 24
Patel et al. (2009)	12	12	12	12	56	58	9/3	9/3	6
Pattyn and De Smet (2008)	250	190	250	190	50	45	N/S	N/S	Min. 36
Ritter and Gioe (1986)	50	50	50	50	62	62	27/23	27/23	Min. 60
Robb et al. (2009)	30	30	30	30	55	67	18/12	12/18	N/S
Silva et al. (2004)	50	40	50	32	47	54	31/19	12/20	N/S
Stulberg et al. (2009)	337	266	337	266	50	53	228/109	165/101	Min. 24
Wagner and Wagner (1996)	35	70	35	70	36	50	4/31	19/51	Min. 34
Witzleb et al. (2006)	111	88	111	74	51	54	N/C	N/C	N/C
Zywiel et al. (2009)	33	33	33	33	53	53	23/10	23/10	Min. 42

A variety of different HRS and THA prostheses were used in the studies reviewed. The most commonly used HRS system was the Birmingham Hip Resurfacing (Smith and Nephew, Warwick, UK), which was used in 15 papers, while the Durom hip resurfacing system (Zimmer, Warsaw, IN) was used in 8 papers and the Conserve Plus (Wright Medical Technology, Arlington, TN) was used in 6 (Table 4). The THA systems used varied considerably (Table 5).

**Table 4.** Summary of the hip resurfacing prostheses used in the studies included in this systematic review

Birmingham Hip Resurfacing system (Smith and Nephew, Warwick, UK)	15
Durom hybrid resurfacing system (Zimmer, Warsaw, IN)	8
Conserve Plus (Wright Medical Technology, Arlington, TN)	6
Cormet MoM (Corin, Cirencester, UK)	4
Tharies prosthesis	2
Indiana Conservative SR	2
McMinn acetabular component and mini stemmed McMinn femoral resurfacing component (Corin Medical Ltd., Gloucestershire, UK)	1
Articular Surface Replacement (DePuy International Ltd., Leeds, UK)	1
Stemless resurfacing system (DePuy, Warsaw, IND)	1
MoM Metasul articulating surfaces prostheses	1
Not stated	2

**Table 5.** Summary of the total hip arthroplasty prostheses used in the studies included in this systematic review

Implant	Frequency
Metasul femoral head and M/L Taper stem (Zimmer, Warsaw, IN)	3
THR (Stryker-Howmedica-Osteonoics) Trident cup and Accolade femoral component (ceramic or cobalt chrome heads on PE liner	2
Trapezoidal-28 THA	2
Cemented CPS stem (Plus Orthopedics, Swindon, UK) and EPF uncemented acetabular component (Plus Orthopedics)	1
Trident Acetabular component and uncemented THA	1
Ultima cemented femoral stem and Duraloc acetabular component (DePuy, Leeds, UK)	1
Uncemented Birmingham Hip Resurfacing acetabular cup and Freeman stem (Finsbury, Surry, UK)	1
Exeter stem and PE acetabular component (Howmedica, London, UK)	1
Axcel THA (Axcel, Cremascoli, Milan, Italy)	1
Summit and Pinnacle uncemented stem (DePuy Orthopaedics Inc, Warsaw, IN) with Marathon PE acetabular (DePuy Orthopaedics Inc.) or Metal acetabulum (Ultamet; DePuy Orthopaedics Inc.)	1
CLS Spotorno grit-blasted titanium uncemented femoral stem and Metasul femoral head (Zimmer, Winterthur, Switerland)	1
Charnley and T-28 prostheses	1
ASR with Corail or SROM stem (DePuy International Ltd., Leeds, UK)	1
CLS femoral stem (Zimmer, Warsaw, IN) and Durom acetabular component	1
Stemmed THR	1
Exeter stem and conteoporary cup (Stryker, Howmedica, Newbury, UK) or Corail stem and ASR cup (DePuy International Ltd., UK)	1
Uncemented Trident Acetabular System and Accolade Femoral Hip System (Stryker-Howmedica-Osteonics, Allendale, NJ)	1
Exeter stem (Stryker, Howmedica, Newbury, UK) and ABG II (Stryker, Howmedica) or Trilogy acetabular component (Zimmer, Warsaw, IN)	1
Stemmed THA	1
PCA stem and System 12 acetabular cup (Stryker-Howmedica-Osteonics, Allendale, NJ)	1
Metasul MoM THR (Sulzer Orthopedics Ltd., Winterthur, Switzerland)	1
Allofit cup (Zimmer, Warsaw, IN)	1
Hybrid THA of Spectron cemented femoral component (Smith and Nephew Orthopaedics, UK), with Triology uncemented acetabular component (Zimmer Ltd., UK)	1
Ancafit CoC THA (Wright Medical, Arlington, TN)	1
Hybrid THA with PE liner	1
Metasul acetabular liner and femoral head (Zimmer, Warsaw, IN)	1
Exeter stem (Stryker, UK) with polyethylene Opera acetabular cup (Smith and Nephew, UK). Cemented procedure	1
CoC Stryker ABC System (Stryker Orthopaedics, Mahwah, NJ)	1
Uncemented - G2 femoral stem and Duraloc or Pinnacle cup (DePuy Orthopedics, Warsaw, IN) for metal on PE implant	1
CLS Spotorno (Zimmer, Warsaw, IN) Allofit acetabular shell (Zimmer), Metasul acetabular PE insert. 28-mm femoral head. Uncemented	1
MoM Cone prosthesis and CLS stem (Protek AG, Berne, Switzerland)	1
MoM THA (Metasul; Sulzer Orthopedics Ltd., Switzerland)	1
Not Stated	5

CoC: ceramic-on-ceramic; MoM: metal-on-metal; PE: polyethylene; THR: total hip replacement.

### Meta-analysis

#### Clinical outcomes

The results of the meta-analysis indicated that there was a statistically significant difference between HRS and THA for a number of clinical outcomes. Functionally, there was a significantly higher WOMAC score (Bellamy et al. 1988) for patients who underwent THA at final follow-up, indicating poorer functional ability (MD = –2.4, CI: –3.9, –0.9; p = 0.001), and better range of motion component of the Harris hip score (HHS) (Harris 1969) (MD = –0.05, CI: –0.1, –0.03; p < 0.001) and overall HHS (MD = 2.5, CI: 1.2, 3.8; p = 0.001) in the HRS cohorts than in the THA cohorts. Significance from CI indicated that more patients who underwent THA had greater difficulty in undertaking a step test task than those who had HRS (RR = 0.3, CI: 0.1, 0.6; p < 0.0014). However, there was no statistically significant difference between THA and HRS cohorts regarding Merle d'Aubigne index (Merle d'Aubigné and Postel 1954), UCLA (Amstutz et al. 1984), Oxford hip score (Dawson et al. 1996), or hop test results (p > 0.05) (Table 6), although these outcomes were assessed with a smaller number of patients than were the WOMAC and HHS assessments.

**Table 6.** Clinical outcomes after meta-analysis

Outcome	Mean difference	p-value	Heterogeneity	
	(95% CI)		I^2^ (%)	Chi^2^
Last reaming required	0.78 (-0.22 – 1.78)	0.1	70	0.07
Mean incision length	6.42 (-0.49 – 15.33)	0.2	94	< 0.001
Duration of operation	13.63 (7.48 – 19.79)	< 0.001	74	0.004
Estimated blood loss	-152 (-305 – -0.5)	< 0.05	78	0.01
Length of hospital stay	-1.44 (-2.34 – -0.55)	0.002	93	< 0.001
Merle d'Aubigne index	-0.08 (-0.23 – 0.07)	0.3	0	0.61
UCLA	0.72 (-0.27 – 1.71)	0.2	90	< 0.001
Short Form-12 (mental)	1.90 (-8.25 – 4.04)	0.1	0	0.68
Short Form-12 (physical)	3.54 (0.60 – 6.48)	0.02	41	0.18
EQ-5D	0.03 (-0.05 – 0.11)	0.5	N/E	N/E
Patient satisfaction	N/E	N/E	N/E	N/E
Patient satisfaction (satisfied/very satisfied)	1.13 (0.94 – 1.35)	0.2	87	0.07
HHS	2.51 (1.24 – 3.77)	< 0.001	28	0.25
HHS (Function)	N/E	N/E	N/E	N/E
WOMAC	-2.41 (-3.88 – -0.94)	0.001	0	0.77
Oxford hip score	-4.13 (-7.41 – -0.86)	0.6	34	0.22
HHS (ROM)	-0.05 (-0.07 – -0.03)	< 0.001	N/E	N/E
Flexion ROM	-0.23 (-3.78 – 3.31)	0.9	0	0.58
Abduction ROM	-0.31 (-2.16 – 1.55)	0.8	0	0.51
Adduction ROM	3.00 (-0.92 – 6.92)	0.1	N/E	N/E
Internal rotation ROM	2.00 (-4.27 – 8.27)	0.5	N/E	N/E
External rotation ROM	1.00 (-3.51 – 5.51)	0.7	N/E	N/E
Total rotation ROM	-3.83 (-17.50 – 9.85)	1.0	79	0.03
Hop test	0.94 (0.16 – 7.53)	0.9	87	0.005
Step test	0.26 (0.12 – 0.55)	< 0.001	0	0.85
Pain **^a^**	-0.14 (-0.35 – 0.06)	0.2	0	0.36
Presence of groin pain	0.30 (0.59 – 5.27)	0.2	39	0.20
Presence of thigh pain	0.48 (0.09 – 2.55)	0.4	N/E	N/E
Frequency of blood transfusion requirement	0.37 (0.23 – 0.61)	< 0.001	0	0.97

**^a^** standardized mean difference. HHS: Harris hip score; N/E: not estimable; ROM: range of motion.

There was a difference regarding Short Form-12 (SF-12) physical component scores (Ware et al. 1996) (MD = 3.5, CI: 0.6, 6.5; p = 0.02), but there was no statistically significant difference between prosthesis groups for SF-12 mental component scores (Ware et al. 1996) and EQ-5D scores (Brooks 1996) (p > 0.05). However, both outcomes had a degree of statistical heterogeneity (Table 6).

There was no statistically significant difference regarding mean incision length, pain scores, presence of groin or thigh pain, and patient satisfaction outcomes between HRS and THA cohorts at final follow-up (p > 0.05). Similarly, there was no significant difference between prostheses regarding range of motion of the hip (p > 0.05; Table 6).

While the results indicated that there was a greater requirement for blood transfusion following THA (RR = 0.4, CI: 0.2, 0.6; p < 0.001), the difference seen with longer operative duration in HRS procedures (MD = 13.6, CI: 7.5, 19.8; p < 0.001), greater estimated blood loss with THA (MD = –152.8, CI: –305.0, –0.5; p < 0.05), and longer hospital stay with THA (MD = –1.4, CI –2.3, –0.6; p = 0.002) should also be viewed with caution, based on the high levels of statistical heterogeneity reported (Table 6). Furthermore, while the outcomes were statistically significantly different, there were no clinically significant differences between the 2 prostheses.

#### Radiological outcomes

The radiological outcomes assessed showed a higher presence of heterotopic ossification (RR = 1.6, CI: 1.2, 2.1; p = 0.006) in HRS cases than in THA cases. There was no statistically significant difference between the 2 prostheses regarding acetabular or femoral offset, leg length, cup height, or for the presence of specific acetabular or femoral radiolucency (p > 0.05) (Tables 7 and 8).

**Table 7.** Radiological outcomes following meta-analysis

Outcome	Mean difference	p-value	Heterogeneity	
	(95% CI)		I^2^ (%)	Chi^2^
Femoral offset	-15.49 (-48.31 – 17.33)	0.4	100	< 0.001
Acetabular cup offset	2.20 (-0.95 – 5.35)	0.2	N/E	N/E
Leg length **^a^**	-0.62 (-1.48 – 0.24)	0.2	93	< 0.001
Cup height	N/E	N/E	N/E	N/E

**^a^** standardized mean difference N/E: not estimable.

**Table 8.** Radiology outcomes (dichotomous) following meta-analysis

Outcome	Risk ratio	p-value	Heterogeneity	
	(95% CI)		I^2^ (%)	Chi^2^
Heterotopic ossification	1.62 (1.23 – 2.14)	< 0.001	16	0.3
Acetabular radiolucency present	1.27 (0.18 – 8.78)	0.8	60	0.1
Femoral radiolucency present	0.72 (0.03 – 19.47)	0.8	82	0.004

#### Complications

**Figure 3.**
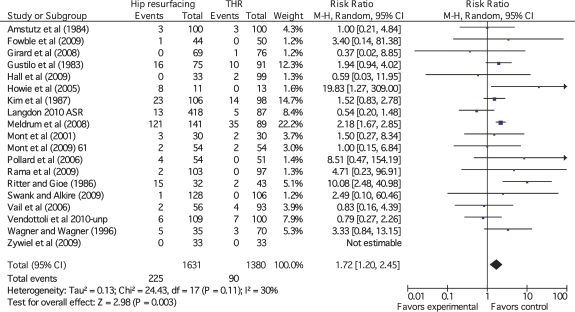
Forest plot to illustrate the difference in frequency of revision surgery between hip resurfacing and total hip replacement.

**Figure 4.**
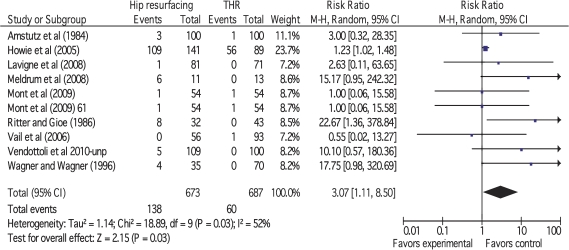
Forest plot to illustrate the difference in frequency of aseptic loosening between hip resurfacing and total hip replacement.

The primary outcome under investigation was the frequency of revision surgery. The risk of revision surgery following HRS compared to conventional THA almost doubled (RR = 1.7, CI: 1.2, 2.5; p = 0.003) (Figure 3). There was also a 3 times greater risk of aseptic loosening in HRS patients than in THR patients (RR = 3.1; 95% CI: 1.1, 8.5; p = 0.03) (Figure 4). These 2 outcomes also showed statistical heterogeneity. There was a reduced incidence of dislocation (RR = 0.2, CI: 0.1, 0.5; p < 0.001) following HRS compared to THA, with no issues of statistical heterogeneity (Table 9).

**Table 9.** Complication outcomes following meta-analysis

Outcome	Risk ratio	p-value	Heterogeneity	
	(95% CI)		I^2^ (%)	Chi^2^
Femoral neck notching	9.2 (2.4–34.7)	0.001	0	0.9
Avascular necrosis	6.8 (1.7–27.6)	0.01	0	0.6
Aseptic loosening	3.1 (1.1–8.5)	0.03	52	0.03
Revision surgery	1.7 (1.2–2.5)	0.003	30	0.1
Positive Trendelenburg sign	1.7 (0.4–7.0)	0.5	0	0.8
Acetabular malposition	1.5 (0.7–3.1)	0.3	0	0.8
Peroneal nerve plasy	1.2 (0.3–5.6)	0.8	0	0.7
Mortality	1.1 (0.1–17.8)	0.9	62	0.1
Clinical leg length discrepancy	1.1 (0.2–7.4)	0.9	29	0.2
Fracture	0.9 (0.5–1.7)	0.8	0	0.7
Squeaking	0.9 (0.04–18.5)	0.9	51	0.2
Trochanteric bursitis	0.9 (0.1–8.1)	0.9	46	0.2
DVT-PE	0.8 (0.3–2.0)	0.6	0	0.7
Trochanteric malunion	0.8 (0.2–2.6)	0.7	0	0.6
Sciatic nerve palsy	0.7 (0.2–3.1)	0.6	0	0.6
Joint infection	0.5 (0.2–2.8)	0.1	0	0.8
Adverse reaction to metal debris failure	0.2 (0.02–2.5)	0.2	62	0.1
Dislocation	0.2 (0.1–0.5)	< 0.001	0	0.9

DVT-PE: Deep vein thrombosis and pulmonary embolism

There was no statistically significant difference regarding the incidence of postoperative fracture, VTE or pulmonary embolism (PE), joint infection, acetabular component malpositioning, trochanteric malunion, peroneal or sciatic nerve palsy, trochanteric bursitis, clinical leg length discrepancy, squeaking, positive Trendelenburg sign, or mortality between HRS and THA cohorts (p > 0.05) (Table 9). There was no statistically significant difference between the frequency of adverse reaction to metal debris between HRS and THA.

#### Critical appraisal outcomes

The CASP review showed that while 44 papers provided a clearly focused question, only 10 were RCTs (Table 10). All studies were undertaken at independent centers, with none conducted in implant inception centers. Of these studies, 9 clearly described the method of randomization, while in 25 studies the authors were able to demonstrate that their groups were comparable at baseline. The study population was clearly defined in 33 studies. Assessor blinding was used in 4 studies, while patients were blinded as to the type of prosthesis in only 2. In 16 studies, the results were analyzed by intention-to-treat methods or it was stated that all who started the study in the respective groups were analyzed according to the initial allocation. A power calculation was used to justify the study sample sizes in 14 studies. 35 studies used inferential statistics to compare outcomes between the groups, but the precision of the results was presented using confidence intervals in only 5. In 35 studies, the authors interpreted their results appropriately and associated their findings to the previous evidence base.

**Table 10.** Summary of the CASP appraisal results

CASP score (maximum 17)	Frequency
0–3	1
4–7	16
8–11	23
12–15	5
16–17	0

## Discussion

Our findings indicate that while functional outcomes following HRS were better or the same as those following THA, there is a higher risk of heterotopic ossification, aseptic loosening, and subsequent early revision surgery for patients who undergo HRS rather than THA. Accordingly, THA appears to be superior to HRS on the basis of the current evidence base. However, the evidence base presented—with a number of methodological inadequacies such as the limited use of power calculations and poor or absent blinding of both patients and assessors—can give rise to assessor bias. The evidence base was also guilty of poorly documenting methods of recruitment, thus permitting allocation or recruitment bias. Regarding these factors, the current evidence base—while being substantial in size—may be questioned with respect to its quality.

Some results obtained from the meta-analysis may have been predictable, due to inherent differences between the 2 designs. For example, the larger head size of the HRS provides greater stability (thus reducing the risk of dislocation), the removal of the femoral head in THA reduces the risk of avascular necrosis of the femoral head, and the need to site the femoral component would predispose the HRS system to show a greater incidence of femoral neck notching, compared to the THA (Table 9). It is noteworthy that Shimmin and Back (2005) suggested that most failures in HRS procedures are due to fractures of the femoral neck, with an approximate incidence of 2%. While the exact mechanism of this complication remains unknown, it has been speculated that such fractures occur due to notching of the femoral neck during surgery, varus placement of the femoral component, or poor bone quality in the neck (Cossey et al. 2005, Shimmin and Back 2005, Deuel et al. 2009). Accordingly, whether the frequency of these complications is then a function of surgical technique or the design of specific prostheses, or whether anatomical variance is a further issue that may provide variation in the incidence of such complications, remains unknown.

There is growing evidence of adverse reactions occurring with metal-on-metal articulations (Bengs et al. 2008). It remains unclear whether this is due to implant design, to bearing congruence associated with malpositioning of implants, or to patient response to metal ions. Some studies have suggested that HRS can lead to higher levels of chromium and cobalt levels than metal-on-metal THA at final follow-up (Hart et al. 2006, Witzleb et al. 2006, Moroni et al. 2008, Daniel et al. 2010, Langdon et al. 2010). If future studies substantiate these findings, greater consideration may be required regarding the appropriateness of metal-on-metal implants in the long term compared to alternative bearings such as ceramic-on-ceramic or metal-on-polyethylene bearings.

Our study shows that the frequency of revision surgery was nearly twice as high in patients who underwent HRS as in those who underwent THA. Some authors have suggested that this may be due to the design of the prostheses. Bengs et al (2008) suggested that this may be caused by, or at least contributed to by a relatively short arc of motion and a predominance of neck-on-cup impingement. However, HRS is a technically demanding procedure. As with unicompartmental knee replacement, surgeons who are unfamiliar with the procedure, or those undertaking a minimally invasive approach, may have a greater potential for technical errors, which may lead to a greater requirement for revision surgery compared to the more commonly undertaken conventional THA (Siebel et al. 2006, Morlock et al. 2008a). Some authors have suggested that there is a considerable learning curve to HRS procedures (Shimmin et al. 2005). Notably, the cup inclination angle may be particularly important regarding optimal positioning to reduce impingement, which could relate to implant failure or asymmetrical bearing wear (Siebel et al. 2006, De Haan et al. 2008, Morlock et al. 2008b).

The design of the HRS preserves femoral bone stock (Crawford et al. 2005, Su et al. 2010). While in this review we found no significant difference in bone removal between the size of the last reamer required to prepare the acetabulum (reiterated in the clinical experiences of Muirhead-Allwood et al. (2006), some authors have reported that substantially greater acetabular bone removal occurs with HRS than in conventional THA (Crawford et al. 2005, Loughead et al. 2006, Naal et al. 2009). In this instance, if acetabular failure were to occur, this would be more challenging to revise in a resurfacing implant compared to a conventional implant, as indicated in recent case series (Cuckler 2006, Sandiford et al. 2008, Taylor et al. 2009, Lachiewicz 2009). Continuing research is required to assess the long-term outcomes of revision of HRS.

Bone mineral density (BMD) was assessed by Kishida et al. (2004) and Hayaushi et al. (2007). They concluded that postoperative BMD was greater in the proximal femur in patients treated with HRS than in those treated with conventional THA, suggesting that the transfer of load to the proximal femur was more physiological after HRS. However, this may be dependent on the design of the conventional THA used, where the stem would transfer the load of the femoral neck more physiologically rather than causing simultaneous stress shielding (Kärrholm et al. 2002, Albanese et al. 2006). Watanbe et al. (2000) conducted a finite-element analysis study of HRS. They reported stress shielding in the anterosuperior region of the femoral neck beneath the prosthesis and stress concentrations around the short stem in the inferior cross section of the femoral neck. These authors suggested that these changes may contribute to fractures of the femoral neck and long-term aseptic loosening, which may support the higher incidence of loosening found in this meta-analysis. Kishida et al (2004) suggested that such fractures are early complications and that atrophy of the femoral neck from stress shielding would occur as a later complication. This is contrary to their findings of BMD presentation in the proximal femur, which reported the distribution of stress after a hip resurfacing as relatively normal (Kishida et al. 2004). Since there was not enough data to allow a meta-analysis of different BMD values between HRS and THA, further studies assessing BMD are required to evaluate these assumptions further.

No studies assessing the cost effectiveness of HRS compared to THA surgery were identified. This is a major issue, given that this study has indicated that HRS is a surgical option for patients who are of working age (mean age 51 years), and there may be further costs associated with a greater incidence of revision surgery compared to THA. McKenzie et al. (2003) assessed the economic effects of both younger and physically active elderly patients with HRS in relation to THA patients. They reported that while a THA was more cost effective, this difference was minimal between the groups. They concluded that there was not enough long-term data to answer this question fully. Further studies have been proposed to address this research question (Achten et al. 2010). Following this and similar studies, it will then be possible to determine the most clinical and cost-effective means of managing younger and physically active patients.

Our study had 3 limitations. Firstly, the objective was to assess whether there was a difference in clinical outcomes between patients who underwent HRS and those who underwent THA. Accordingly, we have therefore not attempted to assess whether there is a difference in outcomes between specific THA or HRS prostheses in the meta-analysis. Secondly, while our study indicated that there may be some small differences in functional outcomes between these 2 designs, it remains unclear whether this is attributable to differences in functional kinematics, as motion analysis studies were also not assessed in this review. Furthermore, it remains unclear whether the small difference in age between the 2 cohorts was an important confounding variable between the 2 prostheses. Finally, recent studies have begun to investigate reasons for HRS failure (Nunley et al. 2009, Yue et al. 2009). These have indicated that age and sex appear to be important prognostic variables (Nunley et al. 2009, Yue et al. 2009, McBryde et al. 2010). It was not possible to perform subgroup analysis to determine whether there was a difference between THR and HRS in this review. Further study is therefore recommended to assess these variables.

### Conclusions

In summary, our findings indicate that functional outcomes following HRS are better or the same as for THA, but that there is an increased risk of heterotopic ossification and aseptic loosening after HRS, and the revision rate with HRS is twice that with THA. THA would therefore appear to be superior to HRS.
